# Cross-neutralizing anti-HIV-1 human single chain variable fragments(scFvs) against CD4 binding site and N332 glycan identified from a recombinant phage library

**DOI:** 10.1038/srep45163

**Published:** 2017-03-23

**Authors:** Lubina Khan, Rajesh Kumar, Ramachandran Thiruvengadam, Hilal Ahmad Parray, Muzamil Ashraf Makhdoomi, Sanjeev Kumar, Heena Aggarwal, Madhav Mohata, Abdul Wahid Hussain, Raksha Das, Raghavan Varadarajan, Jayanta Bhattacharya, Madhu Vajpayee, K. G. Murugavel, Suniti Solomon, Subrata Sinha, Kalpana Luthra

**Affiliations:** 1 Department of Biochemistry, All India Institute of Medical Sciences, New Delhi, India; 2 Molecular Biophysics Unit, Indian Institute of Science, Bangalore, India; 3 HIV Vaccine Translational Research Laboratory, Translational Health Science and Technology Institute, NCR Biotech Science Cluster, Faridabad, Haryana, India; 4 International AIDS Vaccine initiative, USA; 5 Department of Microbiology, All India Institute of Medical Sciences, New Delhi, India; 6 Y.R. Gaitonde Centre for AIDS Research and Education (YRG CARE), Chennai, India; 7 National Brain Research Centre, Manesar, Gurgaon, Haryana, India

## Abstract

More than 50% of HIV-1 infection globally is caused by subtype_C viruses. Majority of the broadly neutralizing antibodies (bnAbs) targeting HIV-1 have been isolated from non-subtype_C infected donors. Mapping the epitope specificities of bnAbs provides useful information for vaccine design. Recombinant antibody technology enables generation of a large repertoire of monoclonals with diverse specificities. We constructed a phage recombinant single chain variable fragment (scFv) library with a diversity of 7.8 × 10^8^ clones, using a novel strategy of pooling peripheral blood mononuclear cells (PBMCs) of six select HIV-1 chronically infected Indian donors whose plasma antibodies exhibited potent cross neutralization efficiency. The library was panned and screened by phage ELISA using trimeric recombinant proteins to identify viral envelope specific clones. Three scFv monoclonals D11, C11 and 1F6 selected from the library cross neutralized subtypes A, B and C viruses at concentrations ranging from 0.09 μg/mL to 100 μg/mL. The D11 and 1F6 scFvs competed with mAbs b12 and VRC01 demonstrating CD4bs specificity, while C11 demonstrated N332 specificity. This is the first study to identify cross neutralizing scFv monoclonals with CD4bs and N332 glycan specificities from India. Cross neutralizing anti-HIV-1 human scFv monoclonals can be potential candidates for passive immunotherapy and for guiding immunogen design.

The high genetic diversity of HIV-1 envelope glycoprotein remains a big challenge for the development of an effective vaccine^1^. A robust immune response, involving development of broadly neutralizing antibodies (bnAbs), is elicited by only about 25% of HIV-1 chronically infected donors^2^,^3^,^4^,^5^. Immunogen design is targeted at eliciting similar bnAbs in the vaccinees as observed during natural infection. For many years, only four first generation bnAbs were known: 2G12^6^, b12^7^, 2F5^8^, and 4E10^9^. After the advent of high throughput technologies like single cell sorting and microneutralization, a number of second generation bnAbs have recently been isolated from different HIV-1 infected donors worldwide, the majority of whom are infected by subtype_ B viruses^10^,^11^,^12^,^13^,^14^. On the basis of their target epitopes, these bnAbs can be divided into five types: those targeting glycan dependent trimeric epitopes in the V2 apex, PG9, PG16^11^; the base of the V3 with associated glycans (V3-glycan), 2G12 and the PGT121 and PGT128 antibody families^15^; the CD4 binding site (CD4bs), b12, 3BNC117^16^ and the VRC01^12^ family of antibodies and a very recent N6^17^ antibody; the trimer specific gp120–gp41 interface bnAbs like PGT151^15^, 35O22^14^; and the gp41 membrane-proximal external region (MPER) exemplified by 10E8^13^ bnAb. So far, very few mAbs have been isolated from subtype_C infected donors^18^,^19^, of which only CAP256 has demonstrated high potency and breadth^20^. More than 50% of the HIV-1 infection globally and more than 90% of the infections in India are due to subtype_C viruses. Also, variability in the envelope of HIV-1 within the same subtype in different populations^21^ identifies the need for extensive characterization of the correlates of protection in HIV-1 infected individuals with different ethnicity, with special attention to HIV-1subtype_C.

Studies in macaques and humanized mice demonstrated that passive immunization with adequate amount of bnAbs before virus challenge reduces the viraemia and confers protective immunity^22^,^23^,^24^,^25^, but their efficacy in humans is yet to be evaluated. A recent phase I clinical trial showed for the first time, that passive infusion of a single CD4 binding site directed bnAb (3BNC117) in humans reduced the viraemia in HIV-1 infected donors efficiently, although for a limited time period^26^. Infusion of VRC01 also showed significant decrease in plasma viremia in a single infusion of the bnAb^27^. Antibodies can also serve as HIV-1 microbicides, by inhibiting mucosal viral entry. Thus bnAbs against HIV-1 are potential candidates for a safe and effective eradication strategy, by way of prevention, immunotherapy and cure.

It has been proven by studies that engineered antibody fragments can be more effective and can neutralize even resistant HIV-1 strains as they are smaller than full length IgGs^28^,^29^,^30^,^31^. The anti-HIV-1 IgGs elicited during natural infection often fail to bind to the virus, as binding with both Fabs is prevented due to the low number and density of spikes on the viral envelope, that may in turn lead to the generation of clade-specific antibodies^32^,^33^. Generation of recombinant antibody fragments using *in vitro* display techniques has therefore gained impetus mostly due to its high throughput efficiency in selection, modification and production of antibodies^34^,^35^,^36^,^37^.

A human PBMC based recombinant phage library is likely to provide a repertoire of diverse antibodies in terms of V, D, J gene usage and provide a new spectrum of antibodies, different from the existing bnAbs. Persistent antigenic stimulation and rapid error prone viral replication leading to several mutations promotes antibody maturation against the multiple conserved epitopes^4^. Hence antiretroviral naïve individuals infected for several years are potential candidates for bnAb isolation. Therefore in this study, we have generated an anti-HIV-1 human scFv phage recombinant library by using a novel strategy of pooling PBMCs from select chronic HIV-1 infected Indian donors and identified scFv (single chain variable fragment) monoclonals against different regions of the viral envelope (both conserved and variable). For screening the library for envelope specific clones we used two recombinant trimeric HIV-1 specific proteins: (1) h-CMP-V1cyc1 gp120 protein^38^ and (2) BG505:SOSIP.664^39^. The h-CMP-V1cyc1-gp120 protein preferentially binds to neutralizing Abs than to non-neutralizing ones^38^. The BG505:SOSIP.664 protein is a soluble Env trimer containing gp120 subunits and most of the ectodomain of gp41^39^. The advantage of using BG505:SOSIP.664 is that it mimics the native form of HIV-1 envelope and that it binds to most of the anti HIV-1 bnAbs generated against major epitopes. This is the first study wherein human scFv monoclonals exhibiting cross neutralizing activity against subtype_C and non subtype_C viruses, with CD4bs and N332A glycan specificities, have been identified from a recombinant phage library constructed using select HIV-1 infected donor PBMCs.

## Results

### Construction of human anti-HIV-1 scFv recombinant phage library and identification of HIV-1 specific scFv monoclonals

**Figure 1:**
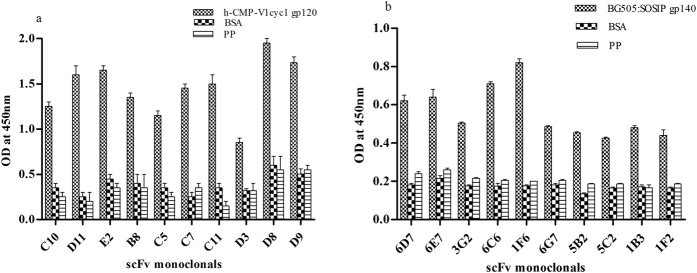
Phage ELISA binding with (**a**) h-CMP-V1cyc1 gp120 and (**b**) BG505:SOSIP.664. scFv clones exhibiting high binding to the h-CMP-V1cyc1 gp120 and BG505:SOSIP.664 were selected. ELISA plates were coated with h-CMP-V1cyc1 gp120, BSA and a peptide pool of unrelated viruses (negative controls). The experiment was repeated at least twice and the mean OD values are shown. Mean binding titres were compared with negative control.

Total RNA was isolated from the pooled PBMCs of six selected HIV-1 infected donors based on their potent viral neutralization efficiency. The plasma neutralization data of four infected donors (AIIMS 617, AIIMS 619, AIIMS 621, and AIIMS 627) recruited from AIIMS and INDO-SA 2007 from YRG Care centre is described elsewhere^40^,^41^, while that of INDO-SA 0030 is unpublished data. PCR analysis of the heavy chain (VH) and light chain genes (VLκ and VLλ) from the cDNA of the pooled PBMCs revealed the amplification of all heavy chain genes except VH3 (Figure S1a) and all light chain genes (Figures S1b and c), each with a band size of approximately 400 base pairs. Pooling of the heavy and light chain amplicons in equal amounts of 100 ng each, followed by assembly PCR (pull through PCR) yielded scFv fragment of 800 bp (Figure S1d). Ligation of the scFv into phagemid vector (pAK100) followed by transformation into TG1 *E. coli* cells led to the generation of the human anti-HIV-1 recombinant phage library. Colony PCR of 10 randomly picked scFv clones (5 clones with λ light chain and 5 clones with κ light chain) from the recombinant phage library confirmed the expression of complete scFv in each of these clones (Figure S2). In order to determine the diversity of the library we randomly picked 57 clones from the unselected library. Amplification of these scFvs was done by PCR, followed by digestion with BstN1^42^. DNA fingerprinting revealed that 45/57 (79%) clones were unique (Figure S3). Further, sequencing of randomly picked scFv clones revealed that 4 out of 5 clones were different (data not shown) thereby establishing the diversity of the library to be comprised of 7.8 × 10^8^ scFv clones. Envelope specific scFv expressing phage clones from the library were selected by four rounds of biopanning with two recombinant trimeric HIV-1 envelope glycoproteins: (1) h-CMP-V1cyc1 gp120 and (2) BG505:SOSIP.664. The binding specificity of around 200 clones randomly selected from both the third and fourth round of panning was assessed in soluble phage ELISA. Ten scFv clones biopanned using each envelope glycoprotein were identified which showed high binding in soluble phage ELISA with the h-CMP-V1cyc1 gp120 (Fig. 1a) and to BG505:SOSIP.664 respectively (Fig. 1b).

The scFv monoclonals which showed >3 fold binding as compared with the negative control were then expressed in soluble form in the periplasmic fraction and purified by column chromatography. The preliminary neutralization activity of all the purified scFv monoclonals was determined with one clade B and one clade C virus (data not shown). Three scFv monoclonals (D11, C11 and 1F6) that demonstrated neutralization activity against one clade B and one clade C virus (data not shown) were further extensively characterized. The expression of the purified scFv monoclonals was analysed by SDS-PAGE (Figure S4c) and confirmed by Western blotting (Figure S4d).

### Determination of the gene usage of the selected scFv confirmed the diversity of the monoclonals

The diversity of the three scFv monoclonals was confirmed by sequence analysis. The gene usage of the three clones was determined using Immunoglobulin BLAST and IMGT/V-Quest software program (Table 1). All the three cross neutralizing scFv monoclonals had distinct sequences with different gene usages. The h-CMP-V1cyc1-gp120 selected scFv monoclonals (D11 and C11) showed VH5–51 heavy chain gene usage and VL6-57 light chain gene usage for D11 and VL3-1 light chain gene usage for C11. The BG505:SOSIP.664 selected scFv monoclonal (1F6) showed VH1-46 heavy gene usage and VL2-14 light chain gene usage. The D11 scFv showed the longest CDRH3 of 19 amino acids whereas CDRH3 of C11 and 1F6 scFvs comprised of 15 and 14 amino acids respectively.

### Cross neutralizing potential observed in the three selected anti-HIV-1 human scFv monoclonals

The HIV-1 neutralization efficiency of C11, D11, and 1F6 scFv monoclonals was checked against a standard panel of 50 pseudoviruses/primary isolates from different subtypes (15 subtype_B, 32 subtype_C, 2 of subtypes_ A and and 1 of subtype_E). The scFv monoclonals were tested at concentrations ranging from 0.09 to 100 μg/ml. The tier 2 viruses 92RW020 (clade A), QZ4589 (subtype_B), JRCSF (subtype_B) and DU422.1 (subtype_C) were neutralized by D11 and C11 scFv monoclonals at significantly low IC50 values. The D11 scFv neutralized 10/16 subtype_C Indian primary isolates tested^43^,^44^,^45^ (mostly known to be resistant viruses). The C11 scFv achieved IC50 titers for 27 viruses, D11 scFv for 33 viruses, 1F6 scFv for 19 viruses, out of the 50 viruses tested (Table 2).

### Affinity measurement, binding and neutralization activity confirmed the specificity of scFv monoclonals

**Figure 2:**
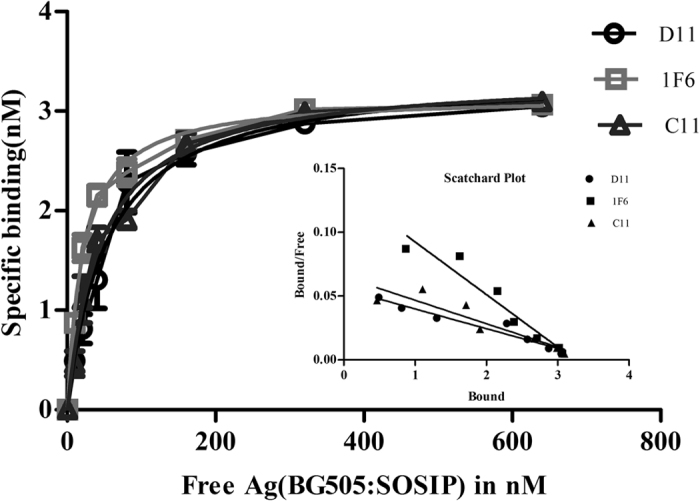
Dissociation constant (*KD*) of each of the 3 scFv-BG505:SOSIP.664 gp140 complex was determined by ELISA. Different dilutions of BG505:SOSIP.664 gp140 (10–640 nM) were incubated with fixed concentration of all the three scFvs (3.125 nM) and unbound antibody was detected by ELISA. The experiment was repeated at least twice. Saturation binding curve was plotted between free antigen Vs specific binding through non-linear regression analysis. KD values for C11 scFv is 4.581 ± 0.5613 × 10^−8^ M (R^2^ = 0.9776), for D11 scFv is 5.362 ± 0.8178 × 10^−8^ M (R^2^ = 0.9676), and for 1F6 scFv is 2.150 ± 0.1921 × 10^−8^ M (R^2^ = 0.9868).

The dissociation of each of the 3 scFv-BG505:SOSIP.664 gp140 complex was determined by ELISA^46^ and saturation binding curve was plotted through non-linear regression analysis^47^ (Fig. 2). Dissociation constant (*K*D) value for C11 scFv is 4.581 ± 0.5613 × 10^−8^ M (R^2^ = 0.9776), for D11 scFv is 5.362 ± 0.8178 × 10^−8^ M (R^2^ = 0.9676), and for 1F6 scFv is 2.150 ± 0.1921 × 10^−8^ M (R^2^ = 0.9868).

**Figure 3:**
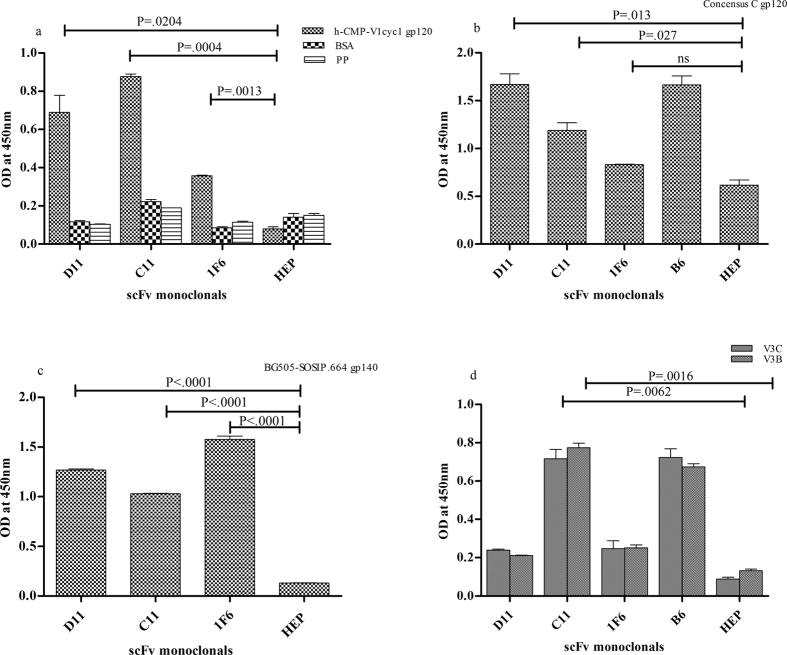
ELISA binding of scFv monoclonals with HIV-1 envelope glycoproteins: ELISA was done to determine binding specificity of the purified scFv monoclonals (at 10 μg/ml concentration) with (**a**) h-CMP-V1cyc1 gp120 (**b**) with consensus C gp120 (**c**) and with trimeric BG505-SOSIP.664 gp140. HEP scFv, the scFv against the hepatitis antigen^48^ served as negative control for all the assays. (**d**) ELISA binding reactivity of scFv monoclonals (at 10 μg/ml concentration) with V3C and V3B peptides was tested. B6, an anti-V3 scFv generated in our lab earlier (unpublished data), is used as positive control. Mean binding titres were compared with negative control using un-paired t test. P values < 0.05 were considered as significant.

The functional activity of the purified C11, D11 and 1F6 scFv monoclonals were assessed by their binding to the h-CMP-V1cyc1 gp120 (Fig. 3a) and BG505:SOSIP.664 trimeric envelope glycoprotein (Fig. 3c). The D11 and C11 alongwith 1F6 scFv monoclonal showed significant binding (P < 0.0001) to BG505:SOSIP.664 gp140 corroborating the ability of neutralizing antibodies to bind BG505:SOSIP.664 gp140 (Fig. 3c). Further, all the 3 scFvs were checked for their binding with concensus C gp120 protein (Fig. 3b). The D11 and C11 scFvs showed significant binding (P = 0.013 and P = 0.027) with consensus C gp120 confirming the binding activity against subtype_C sequences (Fig. 2b). HEP scFv was taken as negative control for scFvs^48^.

The C11 scFv monoclonal showed high binding (P = 0.0016 and 0.0062) to both V3C and V3B peptides suggesting their V3 specificity (Fig. 3d). Epitope mapping of C11 scFv was done by ELISA binding assays using linear overlapping peptides comprising mainly V3 region flanked with second constant (C2) and third constant (C3) region of gp120. The C11 scFv monoclonal showed binding to residues (IRQAHCN) in the V3 base region (Table S1). In order to check the dependency of C11 scFv on N332 in V3 base region, neutralization assays were done with CAP256 wild type and its N332A mutant^49^,^50^,^51^. The N332A mutant showed >two fold increase in IC50 values as compared to its CAP256 wild type virus confirming N332 dependency in the V3 region for C11 scFv monoclonal (Table 3). Further to confirm glycan dependent binding^11^ of C11 scFv monoclonal, ELISA was performed with endo H treated (deglycosylated) and mock treated gp120s (JRFL(subtype_B) and DU422.1(subtype_C)). 2G12 (anti-glycan) and b12 (anti-CD4bs) antibodies were taken as positive and negative controls respectively (Figure S5). The C11 scFv did not bind with deglycosylated gp120, further confirming its glycan dependent binding to the viral envelope.

**Figure 4:**
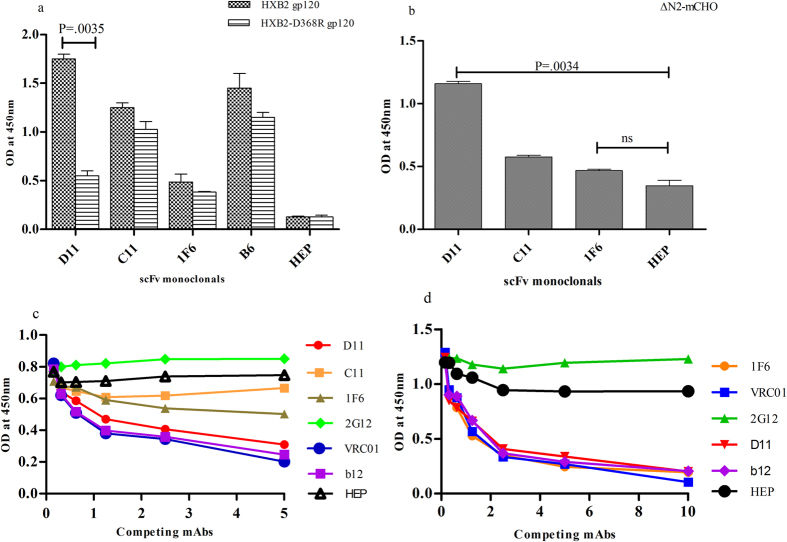
ELISA to determine the epitope specificities of scFv monoclonals. (**a**) Purified scFv monoclonals (at 10 μg/ml concentration) were further checked for binding reactivity with HXB2 gp120 and its D368R mutant by ELISA (**b**) and also their binding with ∆N2-mCHO (at 10 μg/ml concentration) to determine CD4bs specificity. HEP scFv, the scFv against the hepatitis antigen served as negative control for all the assays. Mean binding titres were compared using un-paired t test. P values < 0.05 were considered significant. (**c**) Competition ELISA of biotinylated b12 (at fixed concentration of 50 ng/ml) to RSC3 with decreasing concentrations of scFvs (D11, C11, 1F6 and HEP) and mAbs (2G12, VRC01, b12). 2G12 and HEP scFv were used as negative controls and the bnAb VRC01 was used as positive control for the assay. Dilution used for antibodies is 5 μg/ml to 0.1526 μg/ml. (**d**) Competition ELISA of biotinylated VRC01 (at fixed concentration of 100 ng/ml) to BG505:SOSIP.664 with decreasing concentrations of scFv monoclonals (D11, 1F6 and HEP) and mAbs (2G12, VRC01, b12). The mAb 2G12 and HEP scFv were negative controls and mAb b12 was the positive control in the assay. Dilution used for antibodies is 10 μg/ml to 0.3125 μg/ml.

Next, ELISA binding to HXB2 gp120 and its D368R mutant evaluated specificity of D11 and 1F6 scFv monoclonals (Fig. 4a). Interestingly, D11 and C11 showed high binding to HXB2 gp120 but 1F6 did not show any binding. The D11 scFv monoclonal showed a significant reduction in its binding to HXB2-D368R mutant suggesting its CD4bs specificity (Fig. 4a). In addition, D11 scFv demonstrated binding reactivity with the ∆N2mCHO, a recombinant hyper-glycosylated envelope gp120 (shown to bind CD4bs antibodies^52^,^53^) (Fig. 4b). The CD4bs specificity of D11 and 1F6 scFv monoclonals was confirmed by performing competition ELISA with biotinylated b12 (Fig. 4c) and with biotinylated VRC01 (Fig. 4d) mAbs respectively. Further validation was done by fine mapping the ELISA binding reactivity of D11 and 1F6 scFv monoclonals to linear overlapping peptides (consensus B), spanning the Loop D, CD4 binding loop region (CD4BLP), V5 β24-α5 connection, regions that are known to bind to the CD4bs antibodies^17^,^54^ (Table S2). D11 and 1F6 showed binding to the peptides in CD4BLP confirming their CD4bs specificity. D11 scFv monoclonal showed binding to QSSGGDP peptide in CD4BLP region and to the IVQLNESVEIN peptide of the C2-V3 junctional region of the gp120 whereas IF6 recognized sequences GDPEIVM from CD4BLP and GDMRDNW from V5-β24-α5 connection similar to VRC01 (Table S2).

## Discussion

Broadly neutralizing antibodies (bnAbs) with distinct epitope specificities, have been shown to be effective in blocking HIV-1 infection and controlling viraemia in non-human primates, humanized mice^5^,^23^,^24^,^25^,^55^ and recently in human trials with 3BNC117, providing supportive evidence for the potential role of bnAbs in passive immunotherapy^26^. Further, studying the epitope specificities of these bnAbs can identify candidate epitopes which can be included in a prospective immunogen to improve the breadth of the vaccine protection.

This is the first study to generate a human pooled PBMC based anti-HIV-1 scFv phage recombinant library with diverse antigenic specificities. Earlier, we used a combined approach of EBV immortalization of V3 specific B cells selected from a single HIV-1 infected donor, followed by construction of antigen preselected human anti-V3 recombinant scFv library of limited diversity; the advantage of such antigen (V3) preselected library being the ease of isolating V3 directed scFv monoclonals^42^.

Some of the potent germline antibodies from individual HIV-1 infected donors however are lost during B cell development and evolution due to showing cross reactivity with self-antigens, extensive viral mutations, and evolution of antibodies to immuno-dominant epitopes that are present in the circulating viruses in the infected donor. Hence to obtain scFv antibody fragments with varied and rare combinations of the heavy and light chains that are not often observed in natural infection and moreover cannot be derived from any single infected donor, we have pooled HIV-1 infected human PBMCs from potent neutralizers to attain an antibody library of high diversity. This strategy maximizes the probability of identifying antibodies against the various epitopes on the viral envelope by screening the library with the currently available HIV-1 envelope specific monomers and trimeric proteins^12^,^38^,^39^. Use of the highly selective recombinant trimeric cyclized envelope glycoprotein h-CMP-V1cyc1gp120^38^ and the recent BG505:SOSIP.664gp140^39^ protein enabled the selection and isolation of three cross neutralizing scFv monoclonals (D11, C11 & 1F6). The BG505:SOSIP.664 used in this study is a recombinant envelope glycoprotein constructed from subtype_A virus. Recently, a couple of SOSIP trimers have been constructed from subtype_C viruses ZM197 and DU422^56^. It would be interesting to screen our scFv library with these subtype_C specific SOSIP trimeric envelopes of both African and Indian strains in future as it might yield more potent scFv clones against subtype_C viruses using which newer epitopes can be identified.

Among the 3 scFv monoclonals characterized in this study, D11 demonstrated maximal neutralization efficiency against the peudoviruses of different subtypes and Indian primary isolates. Interestingly, both D11 and C11 exhibited high potency (IC50 < 0.09 μg/mL) in viral neutralization, although against a few viruses. The long CDRH3 of D11, of maximum length among the scFvs studied here, could be one of the plausible contributing factors for the potent cross neutralizing activity exhibited by this scFv. The CD4 binding specificity of D11 and 1F6 was identified by binding assays using linear peptides from the regions recognized by the known CD4bs mAbs b12 and VRCO1 from the CD4BLP^54^ and was further confirmed by competition with b12 and VRC01. The D11 scFv competed with both b12 and VRC01 mAbs whereas 1F6 competed better with VRC01 than b12, suggesting that these two scFv monoclonals partly shared some of the epitopes within the CD4 binding site, a conformational epitope^54^. Moreover, the distinct binding reactivity demonstrated by D11 to the C2-V3 junctional region, which is not observed with the 1F6, suggests that this differential epitope specificity could plausibly be one of the contributing factors for the better neutralization activity of D11 over 1F6. Based on this unique epitope recognized by the D11, further fine mapping by site directed mutagenesis and construction of alanine scanning envelope pseudovirus mutants will enable the identification of novel neutralization determinants for immunogen design. The C11 scFv, showing V3 specificity, demonstrated N332 dependence, a residue seen in most of the subtype C viruses suggesting that this scFv may be effective against majority of the Indian and South African subtype-C viruses^57^,^58^. The limited neutralization breadth shown by this scFv can be overcome by further genetic manipulations of this recombinant antibody fragment.

Majority of the known CD4bs directed bnAbs have been reported with a VH1-2 or VH1-46 gene usage^16^. The D11 scFv monoclonal, with CD4bs directed specificity, and most potent among the three scFvs tested in this study, showed a VH5-51 gene usage. In support of our finding, a recent detailed analysis of CD4bs Abs suggested that the gene repertoire for recognition of the CD4bs region comprises of antibodies with different paratopes that can serve as determining factors for their neutralizing breadth and potency^59^. Similarly in another study^60^, it was shown that NAbs against the CD4bs region can develop by rearrangement from other variable heavy chain genes like VH5-51, rather than VH1-2 or VH1-46 gene. The 1F6 scFv, identified using BG505:SOSIP.664 as antigenic bait, also showed CD4bs specificity, with a VH1-46 heavy chain gene usage and is in consonance with observation that most of the CD4bs antibodies can be gene restricted. The C11 scFv showed N332A glycan specificity within the V3 region, with VH5-51 heavy chain gene usage. It is established that V3 antibodies preferably use this heavy chain gene^61^. Light chain gene usage of all the three scFv monoclonals is distinct.

Neutralizing scFv monoclonals such as D11 can serve as anti-HIV-1 reagents since their small size makes them sterically less hindered and facilitates their easy access to deep seated conserved epitopes in comparison to full length antibodies^31^,^62^,^63^. A recent novel approach has demonstrated success in overcoming the hurdle posed by low density of envelope spikes^32^ by engineering small antibody derivatives and constructing intra-spike crosslinking reagents, which increased the neutralization potency of the engineered antibodies by 2.5 orders of magnitude^33^. To conclude, in this study we have successfully generated human anti-HIV-1 cross neutralizing scFv monoclonal fragments with distinct epitope specificities from Indian infected donors that can serve as potential reagents for blocking HIV-1 infection. Further, combination of antibodies with different epitope specificities can improve the neutralization profile, also combining the antibodies with drugs that can activate latent viruses can be critical for eradication of HIV-1. Thus, by employing antibody engineering strategies, it is feasible to generate bispecific and chemically-modified antibody reagents that can simultaneously target multiple HIV-1 epitopes with high avidity and thereby prevent viral escape.

## Materials and Methods

### HIV-1 positive study participants and blood sample processing

Recruitment of HIV-1 infected donors (AIIMS 617, AIIMS 619, AIIMS 621 and AIIMS 627) and whole blood sampling was done after obtaining ethical approval (IEC/NP-227/2010) for the from the institutional (AIIMS, New Delhi) ethics committee. Experiments were done in accordance with the approved guidelines of the AIIMS ethics committee. In addition, a written informed consent was obtained from all the subjects before enrolment into the study. Plasma was separated and stored at −80 °C. PBMCs from the HIV-1 positive blood samples were separated by Ficoll density gradient method. PBMCs of two of the potential cross neutralizing samples INDO-SA 2007^41^ and INDO-SA 0030 (unpublished data), were obtained from the YRG Care centre, as a part of a muti-centric study (DST/INT/SAFR/Mega-P (4)/2011).

### Antibodies/Vectors

The monoclonal antibodies used in competition experiments in this study (b12, VRC01, 2G12) were obtained from NIH-AIDS Research and Reference Reagent Program (NIH ARRRP). The pAK100 and pAK400 plasmids were kindly gifted by Andreas Pluckthun^64^. For cloning in pAK vector^64^, the *Not1* restriction site in the reverse pull through primer was changed to *Sfi*1 and a new pull through primer was synthesised.

The sequence of the modified Primer (pAK *Sfi1*) is:

5′ TCA GCA T*GG CCC CCG AGG CC*G CAC GTT TRA T 3′

R = A or G

The scFv monoclonals were amplified with a set of forward and reverse primers that contained *Sfi1* site. Upon re-amplification, the scFv and pAK100 vector were digested with *Sfi1* restriction enzyme.

### TZM-bl based Neutralization assay

The neutralization data of the HIV-1 positive samples (AIIMS 617, AIIMS 619, AIIMS 621 and AIIMS 627) has been discussed in detail in our previous study^40^. The neutralization profiling of INDO-SA 2007 is recently published and that of INDO-SA 0030 was unpublished data. Briefly, different dilutions of scFv monoclonals (100 μg/ml–0.09 μg/ml) were incubated with 200 TCID of the pseudoviruses/primary isolates^44^,^45^,^65^ (derived from PBMCs of HIV-1 infected Indian donors) for 1 h at 37 °C. Then TZM-bl cells were trypsinized and seeded at 10^4^ cells/well (in DMEM, containing 25 ug/ml DEAE Dextran). In case of primary isolates, Indianavir (1 mM) was also added to the cells and plates were incubated at 37 °C. After 48 h, luciferase activity was measured (both for pseudoviruses and primary isolates) using the Bright-Glow Luciferase Assay System (Promega Inc.). IC50 values for scFv monoclonals were calculated by a dose-response curve fit with non- linear function, with the help of Graph Pad prism software (San Diego, CA). Each experiment was repeated twice and performed in duplicates and mean IC50 was calculated.

### Construction of human anti-HIV-1 scFv recombinant phage library

10^6^ PBMCs were taken from each of the 6 selected HIV-1 infected donors, and pooled. The procedure adopted for recombinant phage library construction was similar to that reported by us in our previous study^42^; with few modifications. Here, we did not EBV transform the cells or select based on reactivity to any HIV-1 antigen, but instead used the donor PBMCs directly for construction of a phage library. Total RNA was isolated from the pooled PBMCs and reverse transcribed to cDNA. A total of 24 primer combinations^66^ (6 forward primers and 4 reverse primers) were used to amplify heavy chain variable genes from the cDNA. Thirty combinations (6 forward primers and 5 reverse primers) were used to amplify kappa (κ) light chain, and 21 combinations (7 forward primers and 3 reverse primers) were used for lambda (λ) light chain amplification. Full scFvs were amplified through a pull through PCR reaction using Taq DNA polymerase and primers PTfw 5′ CCT TTC TAT GCG GCC CAG CCG GCC ATG GCC 3′ as forward primer and PAK *Sfi*1 5′ TCA GCA T*GG CCC CCG AGG CC*G CAC GTT TRA T 3′ as reverse primer, using conditions described earlier^42^.

### Cloning of scFvs and transformation by electroporation method

The scFv DNA and pAK100 vector were digested and ligated at a 3:1 molar ratio using T4 DNA ligase (New England Biolabs, USA) followed by transformation into electrocompetent *E. coli* TG1 cells by electroporation (conditions: current 25 F, resistance 200 Ohms, voltage 2500 volts). Colonies were pooled into 1 ml of 2XYT medium with glycerol (20%) and stored at −80 °C and liquid nitrogen. The background was determined using 100 ng of digested pAK100 vector simultaneously. In order to determine the diversity of the library we randomly picked 57 clones from the unselected library. Amplification of these scFvs was done by pull through PCR (PTfw 5′ CCT TTC TAT GCG GCC CAG CCG GCC ATG GCC 3′ as forward primer and PAK *Sfi*1 5′ TCA GCA T*GG CCC CCG AGG CC*G CAC GTT TRA T 3′ as reverse primer) as described above, followed by digestion with BstN1^42^,^66^. A phage library of 7.8 × 10^8^ clones was successfully constructed. Macrogen (South Korea) did sequencing of the scFvs commercially using specific primers.

### Panning of the phage library to enrich h-CMP-V1cyc1 gp120 and BG505:SOSIP.664 binding clones

For phage rescue, the phage library was infected with M13-KO7 helper phage, followed by precipitation of phages with PEG/NaCl. Concentration of the phages was determined by titration. We next subjected the phages to four rounds of panning using the recombinant trimeric envelope protein h-CMP-V1cyc1 gp120 and BG505:SOSIP.664 to enrich gp120 and gp140 specific phages respectively. Panning was carried out separately by using h-CMP-V1cyc1 gp120 and BG505:SOSIP.664 coated onto magnetic beads (coating was done as described earlier^40^). The phage clones that bound onto the antigen coated magnetic beads were eluted with 0.2 M glycine (pH 2.2) and were neutralized with 1 M Tris-HCl (pH 9.2). Immediately after elution, phages were used to infect the TG1 cells (OD = 0.5) and the cells were pelleted and plated on chloramphenicol (30 mg/ml) containing 2XYT agar and kept at 37 °C overnight. Colonies were inoculated, grown and glycerol stock was made and stored at −70 °C. This procedure was repeated three times to complete 4 rounds of panning using both the recombinant proteins.

### Phage rescue and phage ELISA

The ELISA plates were coated with 100 μl/well (1 ug/ml) of h-CMP-V1cyc1 gp120, BG505:SOSIP.664, BSA, unrelated antigens in 0.1 M NaHCO_3_ (pH 9.6) buffer and incubated overnight at 4 °C. The ELISAs were performed as described previously^42^.

### Colony PCR amplification and Sequencing

To confirm the presence of complete scFvs in the phage library, 10 clones were randomly picked and plasmid was isolated. The scFv fragments were PCR amplified using forward primer; PTfw 5′ CCT TTC TAT GCG GCC CAG CCG GCC ATG GCC 3′ and reverse primer; (pAK Sfi1) 5′ TCA GCA T*GG CCC CCG AGG CC*G CAC GTT TRAT 3′ under conditions described earlier^42^. DNA fingerprint analysis of randomly selected scFv clones by BstN1 was done as described. Selected scFvs were sequenced commercially and the sequences were analysed for their gene usage and diversity using immunoglobulin BLAST^67^ and IMGT/V-Quest software.

### Expression of soluble scFv and its purification

ScFv phage clones that showed high binding in phage ELISA were processed further for soluble scFv expression. The pAK100 vector carrying scFv DNA fragment and pAK400 vector were digested with *Sfi*I restriction enzyme (New England Biolabs, USA). The scFv DNA was then ligated into pAK400 vector at a 3:1 molar ratio, using T4 DNA ligase (New England Biolabs, USA) and then transformed into *E. coli* HB2151. Colonies were picked and glycerol stocks were stored. These transformed cells were again cultured and induced by 1 mM IPTG, and scFv was purified from the periplasmic extract by Ni-NTA (Qiagen) affinity chromatography as described earlier^42^. The scFv was eluted with elution buffer (50 mM NaH_2_PO_4_, 300 mM NaCl, 300 mM imidazole-pH-8.0). The eluted scFv protein was extensively dialysed against ice cold 1X PBS (pH 7.4), concentrated using ultrafiltration columns (Amicon) with a 10 kDa cut off, filtered through 0.2 μm sterile syringe filter and snap frozen in liquid nitrogen and stored.

### SDS-PAGE and Western Blot

SDS-PAGE and Western blotting were performed as described earlier^42^. Resolving the scFv on 10% SDS-PAGE followed by Western blotting, the scFv was detected using anti-His tag antibody raised in mouse (Sigma) at a 1:1000 dilution in PBST. Secondary anti-mouse HRP (1:3000 dilutions) antibody was added and incubated at RT for 2 h. Each step was followed by extensive washings and colour was developed with DAB (Sigma) as the substrate.

### ELISAs to confirm binding specificity of scFv

ELISAs were performed to check the specificity of the purified scFv by using HIV-1 envelope glycoproteins (h-CMP-V1cyc1 gp120^38^, consensus C gp120, BG505:SOSIP.664 gp140^39^, ∆N2-mCHO^52^,^53^, V3B, V3C, HXB2 gp120 and its D368R mutant and all overlapping peptides). A set of linear overlapping peptides (each 15mer with an 11 amino acid overlap or a 4 amino acid walk) corresponding to the sequence of consensus subtype-B V3 region, CD4 binding loop region, loop D, and V5 β24-α5 region of the gp120, were obtained from the NIH AIDS Research and Reference Reagent Program (NIH, ARRRP). All proteins were coated on ELISA plates at 2 μg/ml and kept overnight at 4 °C followed by blocking of non-specific sites. Next, 100 μl/well of purified scFv at different dilutions (10 μg/ml to 1.25 μg/ml) was added and incubated at 37 °C for 1 h, followed by incubation with primary anti-His tag antibody (raised in mouse, Sigma) at 1:1000 dilution and with secondary anti-mouse HRP conjugated antibody (Sigma) at 1:2000 dilution. Each step was followed by extensive washings. The TMB substrate (Bio-Legend) 100 μl/well was added and incubated at RT till the colour developed. Reaction was stopped by adding 8 N H_2_SO_4_. Absorbance was read at 450 nm.

Deglycosylation of gp120s were done by using Endoglycosidase H^11^. Du422.1 gp120 and JRFL gp120 were treated with 40 mU/μg Endoglycosidase H (Endo H; NEB P0702S) in sodium acetate buffer for 16 hr at 37 °C. Mock treated gp120s were treated under same conditions, but the enzyme was omitted from the reaction. Binding ELISA was performed with both the deglycosylated gp120s as described above.

For competitive ELISA, plates were coated with 1 μg/ml of a sheep anti-gp120 C5 antibody, D7324 (Aalto, Biosciences Ireland). The RSC3 protein at 2 μg/ml, was added at 100 μl/well and kept at 37 °C for 2 h. After blocking, serial dilutions of the competitor antibodies (5 μg/ml to 0.1526 μg/ml) were added to the captured RSC3 in 50 μl plain RPMI. Subsequently, 50 μl of biotin-labelled b12 mAb was added at a final concentration of 50ng/ml. The plates were incubated at 37 °C for 1 h followed by incubation with secondary streptavidin-HRP (Sigma) at 250 ng/ml at 37 °C for 30 min. Each step was followed by extensive washings. Colour was developed with TMB substrate (Bio-legend). Reaction was stopped by adding 8 N H_2_SO_4_. Absorbance was read at 450 nm. In case of biotin labelled VRC01 competition ELISA, BG505:SOSIP.664 was coated at 2 μg/ml and serial dilutions of competitor antibodies were used at 10 μg/ml to 0.3125 μg/ml, followed by the same protocol as described above.

Dissociation constant (*K*D) value of each of the 3 scFv-BG505:SOSIP.664 gp140 complex was determined by ELISA as described elsewhere^46^. Different dilutions of BG505:SOSIP.664 gp140 (10–640 nM) were incubated with fixed concentration of all the three scFv’s (3.125 nM) for 16 h at 4 °C, so that equilibrium was attained. Next day, 100 μl of such equilibrated solution was incubated with BG505:SOSIP.664 gp140 coated ELISA plates (500 ng/per well) for 20 min at room temperature to capture free scFvs. Bound scFv was detected with primary anti-His tag antibody (raised in mouse, Sigma) at 1:1000 dilution and with secondary anti-mouse HRP conjugated antibody (Sigma) at 1:2000 dilution as described before. Saturation binding curve was plotted using non-linear regression method and KD values were calculated.

### Statistical Analyses

Statistical analyses were performed using Graph Pad Prism 5. Non-linear regression curve straight line was plotted using the method of least squares to determine the Max50 and IC50 values. Mean Max50 binding titres were compared using unpaired t test. P values < 0.05 were considered significant.

## Additional Information

**How to cite this article:** Khan, L. *et al*. Cross-neutralizing anti-HIV-1 human single chain variable fragments(scFvs) against CD4 binding site and N332 glycan identified from a recombinant phage library. *Sci. Rep.* **7**, 45163; doi: 10.1038/srep45163 (2017).

**Publisher's note:** Springer Nature remains neutral with regard to jurisdictional claims in published maps and institutional affiliations.

## Supplementary Material

Supplementary Information

## Figures and Tables

**Table 1:** Gene usage of selected cross neutralizing scFv monoclonals.

scFv ID	VH	DH	JH	CDRH3	VL	JL	CDRL3
**D11**	IGHV5-51*01	IGHD2-21*02	IGHJ4*02	CAKGLAYCGGDCYPYFDYW	IGLV6-57*01	IGLJ3*02	CQSYDSSNQVF
**C11**	IGHV5-51*01	IGHD4-11*01	IGHJ5*02	CARQGGNDYSLFDPW	IGLV3-1*01	IGLJ1*01	CRAWDTSTALYSF
**1F6**	IGHV1-46*01	IGHD5*01	IGHJ5*01	CARAPRGYSYLDVW	IGLV2-14	IGLJ1*01	CSSYRSGRTQVF

Heavy and light chain gene usage results of the selected scFv monoclonals. 3 scFv monoclonals D11, C11 and 1F6 were found to have different CDRH3 and gene usage.

**Table 2:** Cross neutralization efficiency (IC50 ug/ml) of h-CMP-V1cyc1 gp120 and BG505:SOSIP.664 selected scFv monoclonals.

Viruses	Tier/Subtype	Origin	C11	D11	1F6
MuLV			>100	>100	>100
92RW020	2A	Rwanda	<0.09	<0.09	37.3
BG505	2A	USA	34.8	49.8	31.8
JRCSF	1B	USA	7.75	5.75	>100
SF162	1B	USA	42.8	<0.09	9.75
BAL.01	1B	USA	>100	>100	>100
QZ4589	2B	Trinidad	<0.09	<0.09	52.3
SC422661.8	2B	Trinidad	22.8	29.3	69.8
TRO11	2B	Italy	4.25	>100	7.25
JRFL	2B	USA	32.3	7.25	>100
RHPA4259.7	2B	USA	3.25	22.8	30.8
REJO4541.67	2B	USA	63.3	75.8	70.8
WITO4160.33	2B	USA	>100	>100	>100
CAAN5342.A2	2B	USA	>100	40.8	42.3
AC10.0.29	2B	USA	>100	40.8	80.8
THRO4156.18	2B	USA	>100	43.8	>100
6535.3	2B	USA	>100	>100	>100
PVO.4	3B	Italy	42.3	39.3	>100
MW965.26	1C	Malawi	>100	>100	>100
25711-2.4	1C	India	35.3	32.3	41.9
25710-2.43	1C	India	78.3	37.8	50.3
16055-2.3	2C	India	52.8	26.3	>100
16936-2.21	2C	India	>100	>100	25.6
001428-2.42	2C	India	60.8	5.75	62.3
AIIMS254	2C	India	>100	21.8	57.3
AIIMS253	nd	India	>100	>100	>100
AIIMS212	2C	India	>100	50.3	>100
AIIMS201	2C	India	>100	>100	>100
AIIMS65	2C	India	>100	24.3	>100
AIIMS126	2C	India	>100	29.3	>100
AIIMS70	2C	India	>100	>100	>100
AIIMS 261	2C	India	47.8	36.8	>100
AIIMS329	2C	India	35.8	37.8	>100
AIIMS 346	2C	India	15.6	>100	>100
AIIMS 355	2C	India	26.3	26.8	>100
AIIMS 504	2C	India	>100	35.3	>100
AIIMS 506	2C	India	>100	>100	>100
AIIMS 511	2C	India	37.8	39.8	>100
AIIMS 519	2C	India	36.8	70.8	>100
AIIMS 529	2C	India	41.8	>100	>100
ZM53M.PB12	2C	Zambia	>100	>100	>100
ZM233M.PB6	2C	Zambia	46.3	38.8	40.8
ZM249M.PL1	2C	Zambia	55.8	48.3	81.3
ZM135M.PL10a	2C	Zambia	>100	>100	>100
ZM214M.PL15	2C	Zambia	>100	48.8	46.3
ZM109F.PB4	1C	Zambia	>100	>100	>100
DU172.17	2C	South Africa	63.3	29.8	>100
DU422.1	2C	South Africa	12.3	4.75	4.75
DU156.12	2C	South Africa	>100	>100	>100
CAP210.2.00.E8	2C	South Africa	76.8	49.8	>100
THA092.009	E	Thailand	>100	>100	nd

Neutralization profile of the three scFv monoclonals: scFv monoclonals were checked for their neutralizing efficiency with a standard panel of subtype_A, B and C pseudoviruses and primary isolates. scFv monoclonal fragments were tested at concentrations ranging from 0.09 μg/ml to 100 μg/ml. HEP scFv^48^ was taken as negative control for scFvs. MuLV was used as a negative control along with virus controls and cell controls. nd: not determined. Non-linear regression curve straight line was plotted using the method of least squares to determine the IC50 values.

**Table 3:** N332A glycan directed neutralization activity of C11 scFv.

Viruses	Neutralization Titers IC50(ug/ml)	Fold IC50 (ug/ml) increase relative to wild type				
	C11	1F6	D11	C11	1F6	D11
**CAP256-WT**	20.8	>50	42.73	—	—	—
**CAP256-N332A**	>50	>50	35.38	>2.4	—	0.8279

N332A dependent neutralization activity (IC50 values) of scFv monoclonals. The scFv monoclonals were checked for their neutralizing efficiency with virus CAP256 wild type and its N332A mutant^50^,^51^, at concentrations ranging from 50 μg/ml to 3.125 μg/ml. HEP scFv^48^ was taken as negative control. MuLV was used as a negative control along with virus controls and cell controls. Non-linear regression curve straight line was plotted using the method of least squares to determine the IC50 values.
