# Supplementary material for: Cross-neutralizing anti-HIV-1 human single chain variable fragments(scFvs) against CD4 binding site and N332 glycan identified from a recombinant phage library
Source: Sci Rep. 2017 Mar 23;7:45163. doi: 10.1038/srep45163 (PMC5362912; doi:10.1038/srep45163)
Supplement: Supplementary Information [file srep45163-s1.pdf]

Cross-neutralizing anti-HIV-1 human single chain variable fragments(scFvs) against CD4 binding site and N332 glycan identified from a recombinant phage library

Lubina Khan[1#], Rajesh Kumar[1#], Ramachandran Thiruvengadam[1#], Hilal Ahmad Parray[1], Muzamil Ashraf Makhdoomi[1], Sanjeev Kumar[1], Heena Aggarwal[1], Madhav Mohata[1], Abdul Wahid Hussain[1], Raksha Das[2], Raghavan Varadarajan[2], Jayanta Bhattacharya[3], Madhu Vajpayee[4], KG Murugavel[5], Suniti Solomon[5], Subrata Sinha[6] and Kalpana Luthra[1]*

Supplementary Data

Cross neutralizing anti-CD4 binding site and anti-V3 human single chain variable fragment (scFv) monoclonals identified from a recombinant phage library constructed from pooled PBMCs

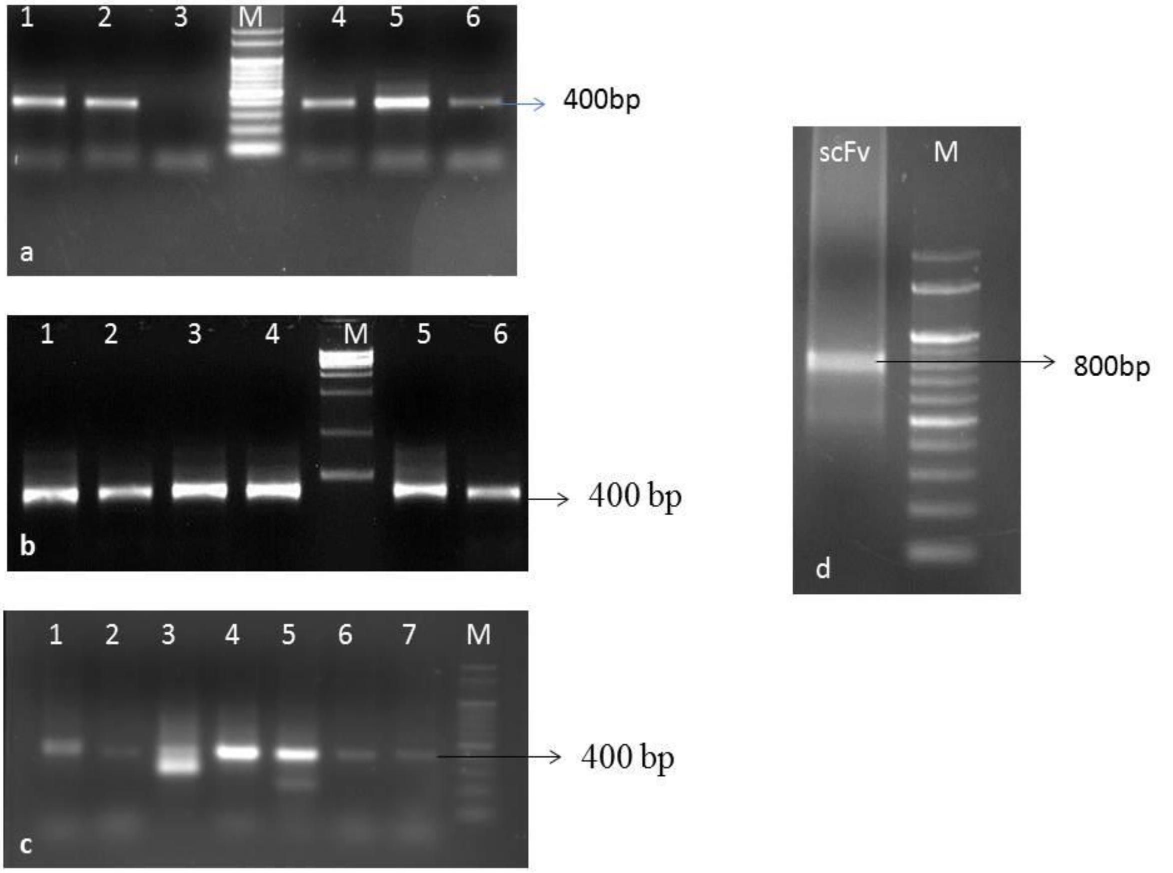

Figure S1. **Amplification of Variable heavy chain genes (VH) and Variable light chain genes(VL):** Amplified PCR products run on 1.5% agarose gel electrophoresis. **a**. Amplified VH genes of 400bp. Lanes 1-6 are amplified VH1, VH2, VH3, VH4, VH5 and VH6 genes respectively, Lane M- 1kb DNA ladder. **b**. Amplified VL kappa genes of 400bp. Lanes 1-6 are amplified VLκ1, VLκ2, VLκ3, VLκ4, VLκ5 and VLκ6 genes respectively, Lane M- 100bp DNA ladder. **c**. Amplified VL lambda genes of 400bp.Lanes 1-7 are amplified VLλ1, VLλ2, VLλ3, VLλ4, VLλ5, VLλ6 and VLλ7 genes respectively, Lane M-100bp DNA ladder. **d.** 800bp scFv fragment amplified by pull through PCR. Lane M, 100bp DNA ladder.

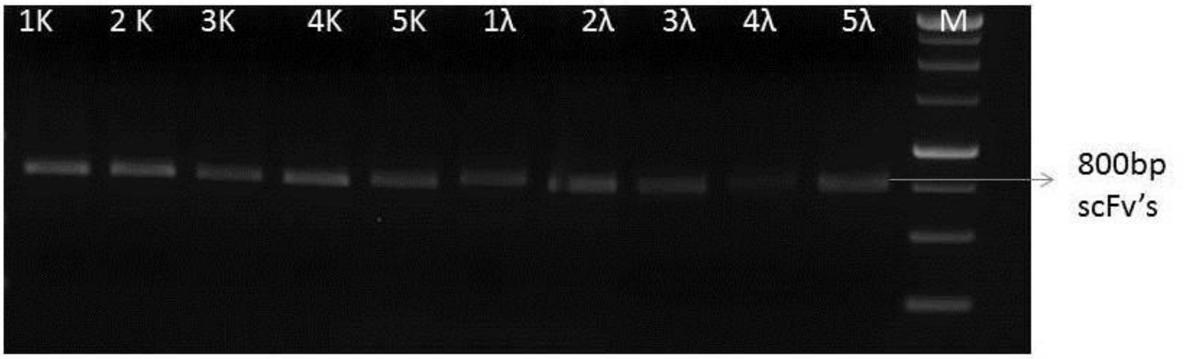

Figure S2. Colony PCR analysis : 1.5% Agarose gel electrophoresis of colony PCR products of 10 randomly selected clones containing kappa and lambda light chain from unscreened phage library. Lane M-1kb bp DNA ladder and Lanes 1K-5K are randomly picked 5 kappa clones. Lanes 1λ-5λ are randomly picked 5 lamda clones

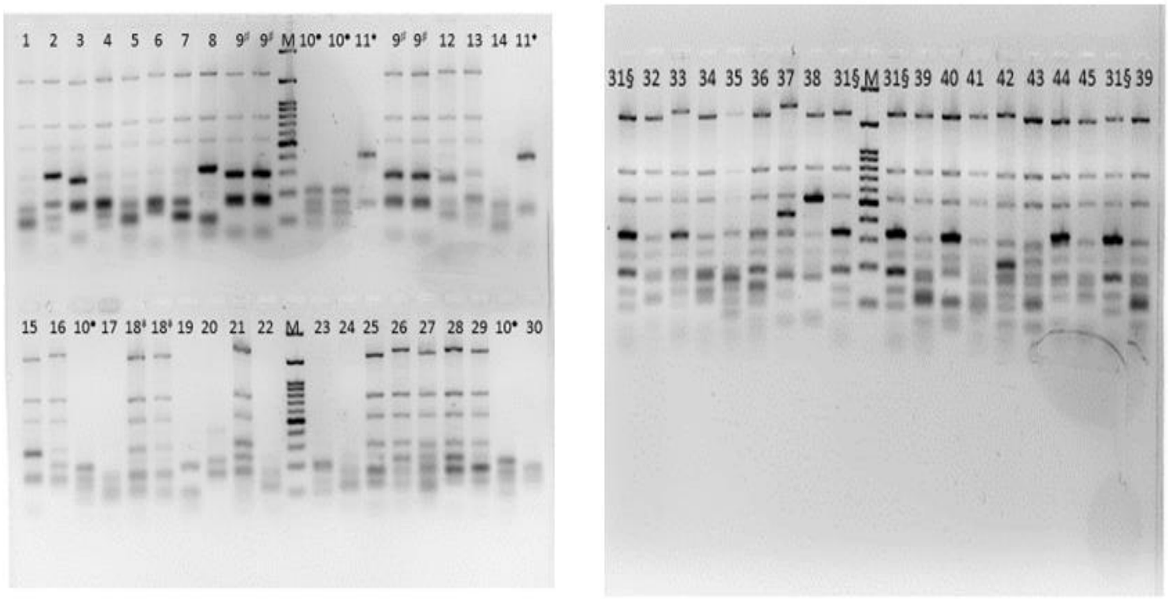

Figure S3. DNA fingerprinting analysis of scFv clones. 57 scFv gene fragments were amplified by PCR and digested with BstN1 at 60°C for 3 hours. Restriction pattern was analysed on 2% agarose gel. 45 /57 clones showed unique DNA fingerprinting pattern. DNA fingerprinting patterns of similar scFvs were assigned same number and symbol. Lane M, DNA ladder (100 bp).

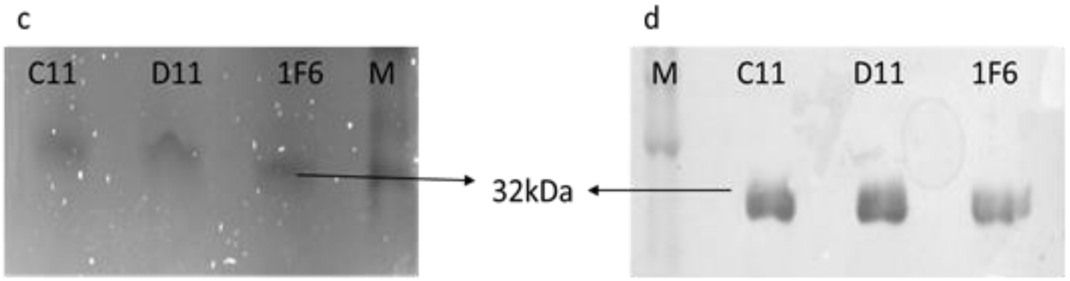

Figure S4. SDS-PAGE and Western blot: (S4c). SDS- PAGE of purified D11, C11, and 1F6 scFvs. Lane M, protein marker. **(S4d)** Western blot analysis of purified scFvs, Lane M, prestained molecular weight marker. ; Arrow indicates the 32kDa band of scFv

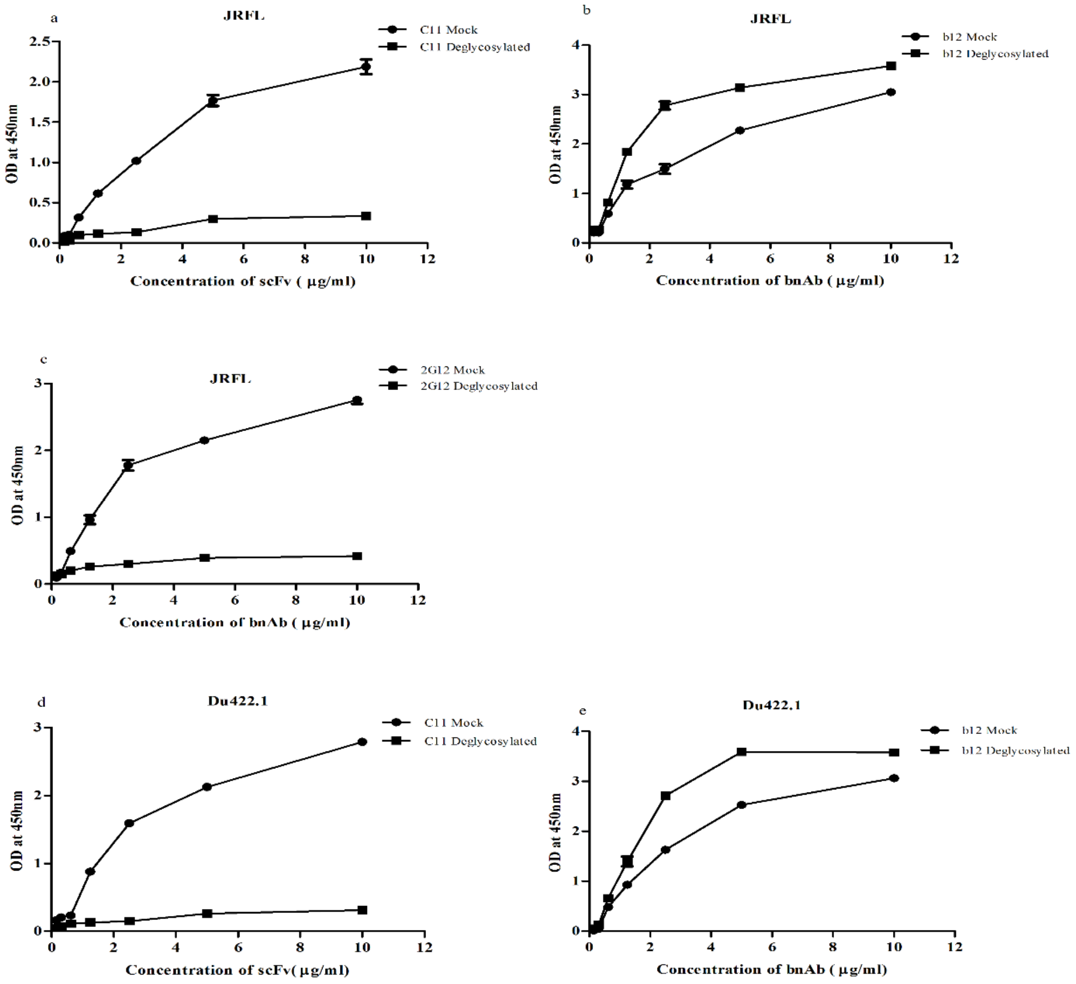

Figure S5. **Binding of C11 scFv monoclonal, b12 (anti-CD4bs), and 2G12 (anti-glycan) to Endo H treated and mock treated gp120s (JRFL and DU422.1) as determined by ELISA.** Binding of 2G12 to gp120Du422 could not be determined because 2G12 does not bind to this isolate.

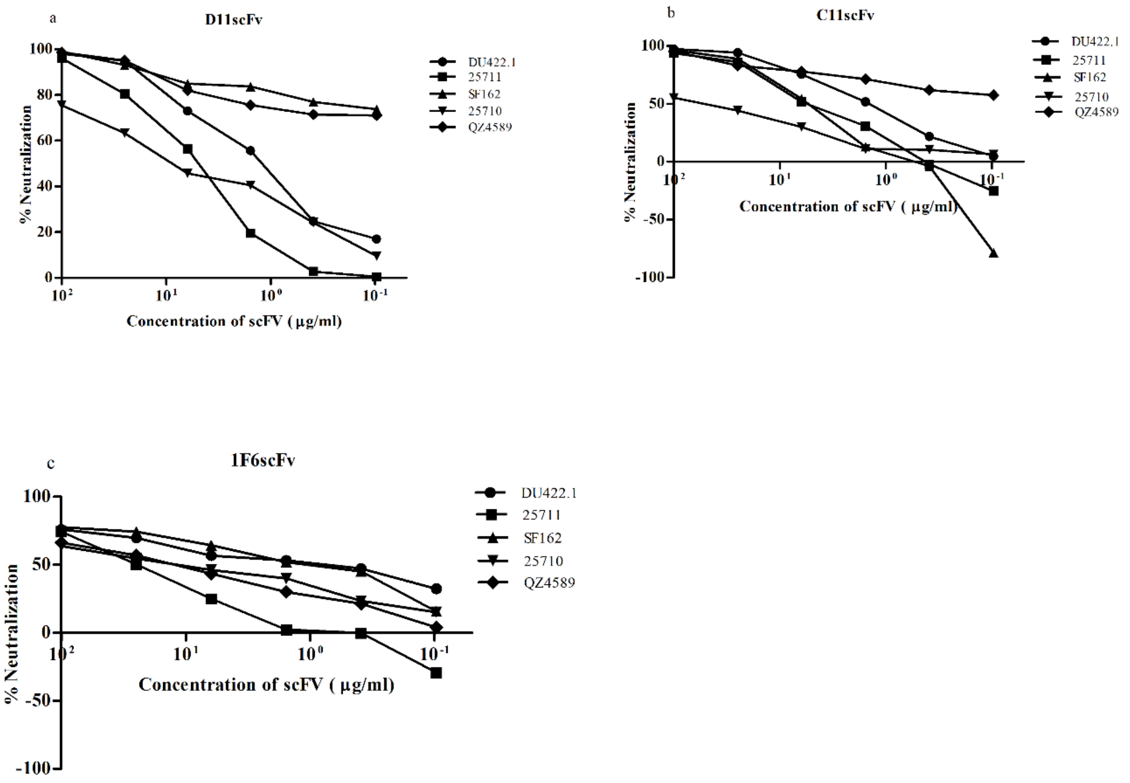

Figure S6. Neutralization curves of scFv monoclonals with representative viruses.

Table S1. Overlapping peptide ELISA binding to determine epitope specificity of scFv monoclonals in concensus B V3 region

PeptideID	Sequence V3 Region	C11	D11	1F6	447-52D	1418
**8834**	IVQLNESVEINCTRP	**>10**	***8.28***	**>10**	**>10**	**>10**
**8835**	NESVEINCTRPNNNT	**>10**	**>10**	**>10**	**>10**	**>10**
**8836**	EINCTRPNNNTRKSI	**>10**	**>10**	**>10**	**>10**	**>10**
**8837**	TRPNNNTRKSIHIGP	**>10**	**>10**	**>10**	***2.08***	**>10**
**8838**	NNTRKSIHIGPGRAF	**>10**	**>10**	**>10**	***4.53***	**>10**
**8839**	KSIHIGPGRAFYTTG	**>10**	**>10**	**>10**	**>10**	**>10**
**8840**	IGPGRAFYTTGEIIG	**>10**	**>10**	**>10**	**>10**	**>10**
**8841**	RAFYTTGEIIGDIRQ	**>10**	**>10**	**>10**	**>10**	**>10**
**8842**	TTGEIIGDIRQAHCN	***7.18***	**>10**	**>10**	**>10**	**>10**
**8843**	IIGDIRQAHCNISRA	***5.23***	**>10**	**>10**	**>10**	**>10**
**8844**	IRQAHCNISRAKWNN	***9.13***	**>10**	**>10**	**>10**	**>10**
**8845**	HCNISRAKWNNTLKQ	**>10**	**>10**	**>10**	**>10**	**>10**

Table S1.Amino acid sequences of linear overlapping peptides of the third variable region flanked by second (C2) and third (C3) constant regions of consensus-B gp120 sequence (from peptide ID 8834 to 8845 from NIH Aids reference and reagent program).C11, D11 and 1F6 scFv monoclonals and 447-52D (positive control) were tested for their binding to overlapping peptides by ELISA using mAbs at a concentration ranging from 10 to 0.3125 µg/ml. The 50% binding titers (Max50, conc. µg/ml) of each antibody against the corresponding peptides is depicted as numerical values in Bold Italic and >10, where Max50 value was not reached. Non-linear regression curve straight line was plotted using the method of least squares to determine the Max50. Human anti-parvovirus B19 mAb 1418 was used as negative control. Each experiment was performed at least two independent times.

Table S2. Overlapping peptide ELISA binding to determine CD4 binding site epitope specificity of scFv monoclonals in CD4 Binding loop (CD4BLP) region, Loop D region and V5-β24-α5 region of concensus B gp120

PeptideID	Sequence CD4BLP	D11 Red	1F6 Green	VRC01	B12	1418
**8850**	REQFGNKTIVFNQSS	>10	>10	>10	>10	>10
**8851**	GNKTIVFNQSSGGDP	*8.28*	>10	>10	>10	>10
**8852**	IVFNQSSGGDPEIVM	*5.23*	*6.38*	*0.975*	*1.93*	>10
**8853**	QSSGGDPEIVMHSFN	*5.88*	*5.68*	*1.63*	*2.48*	>10
**8854**	GDPEIVMHSFNCGGE	>10	*8.38*	*6.23*	*5.33*	>10
**8855**	IVMHSFNCGGEFFYC	>10	>10	>10	>10	>10
**PeptideID**	**Sequence Loop D**	**D11**	**1F6**	**VRC01**	**B12**	**1418**
**8827**	TQLLLNGSLAEEEVV	>10	>10	>10	>10	>10
**8828**	LNGSLAEEEVVIRSE	>10	>10	>10	>10	>10
**8829**	LAEEEVVIRSENFTN	>10	>10	>10	>10	>10
**8830**	EVVIRSENFTNNAKT	>10	>10	>10	>10	>10
**8831**	RSENFTNNAKTIIVQ	>10	>10	*7.68*	>10	>10
**8832**	FTNNAKTIIVQLNES	>10	>10	*8.53*	>10	>10
**8833**	AKTIIVQLNESVEIN	*6.08*	>10	>10	>10	>10
**PeptideID**	**Sequence V5-β24-α5 Region**	**D11**	**1F6**	**VRC01**	**B12**	**1418**
**8876**	GGNNNTNETEIFRPG	>10	>10	>10	>10	>10

**8877**	NTNETEIFRPGGGDM	**>10**	**>10**	**>10**	**>10**	**>10**
**8878**	TEIFRPGGGDMRDNW	**>10**	***6.28***	***3.08***	**>10**	**>10**
**8879**	RPGGGDMRDNWRSEL	**>10**	***5.93***	***3.83***	**>10**	**>10**
**8880**	GDMRDNWRSELYKYK	**>10**	***8.88***	**>10**	**>10**	**>10**
**8881**	DNWRSELYKYKVVKI	**>10**	**>10**	**>10**	**>10**	**>10**
**8882**	SELYKYKVVKIEPLG	**>10**	**>10**	**>10**	**>10**	**>10**

Table S2.Amino acid sequences of linear overlapping peptides of the CD4 Binding loop (CD4BLP) (from peptide ID 8850 to 8855 from NIH Aids reference and reagent program) region, Loop D region (from peptide ID 8827 to 8833 from NIH Aids reference and reagent program) and V5-β24-α5 region (from peptide ID 8876 to 8882 from NIH Aids reference and reagent program) of concensus B gp120. D11 and 1F6 scFv monoclonals and VRC01 and b12 antibodies (positive control) were tested for their binding to overlapping peptides by ELISA at a concentration ranging from 10 to 0.3125 µg/ml. The 50% binding titers (Max50, conc. µg/ml) of each antibody against the corresponding peptides is depicted as numerical values in ***Bold Italic*** and >10, where Max50 value was not reached. Non-linear regression curve straight line was plotted using the method of least squares to determine the Max50 Human anti-parvovirus B19 mAb 1418 was used as negative control. Each experiment was performed at least two independent times
